# An Eccentricity Gradient Reversal across High-Level Visual Cortex

**DOI:** 10.1523/JNEUROSCI.0809-24.2024

**Published:** 2024-11-08

**Authors:** Edan Daniel-Hertz, Jewelia K. Yao, Sidney Gregorek, Patricia M. Hoyos, Jesse Gomez

**Affiliations:** Princeton University, Princeton Neuroscience Institute, Princeton, New Jersey 08544

**Keywords:** center periphery, eccentricity, high-level visual cortex, limb-selective region, receptive field mapping, VTC

## Abstract

Human visual cortex contains regions selectively involved in perceiving and recognizing ecologically important visual stimuli such as people and places. Located in the ventral temporal lobe, these regions are organized consistently relative to cortical folding, a phenomenon thought to be inherited from how centrally or peripherally these stimuli are viewed with the retina. While this eccentricity theory of visual cortex has been one of the best descriptions of its functional organization, whether or not it accurately describes visual processing in all category-selective regions is not yet clear. Through a combination of behavioral and functional MRI measurements in 27 participants (17 females), we demonstrate that a limb-selective region neighboring well-studied face-selective regions shows tuning for the visual periphery in a cortical region originally thought to be centrally biased. We demonstrate that the spatial computations performed by the limb-selective region are consistent with visual experience and in doing so, make the novel observation that there may in fact be two eccentricity gradients, forming an eccentricity reversal across high-level visual cortex. These data expand the current theory of cortical organization to provide a unifying principle that explains the broad functional features of many visual regions, showing that viewing experience interacts with innate wiring principles to drive the location of cortical specialization.

## Significance Statement

What is the organizing principle of high-level visual cortex? Visual stimuli experienced extensively during childhood, like faces or scenes, give rise to specialized regions in visual cortex. These regions emerge in consistent locations across individuals, thought to result from the retinotopic input of earlier visual cortex. The field has quantified this input as a medial-lateral gradient of retinotopic eccentricity in ventrotemporal cortex that has not yet been mapped beyond the fusiform gyrus. By performing receptive field mapping in limb-selective cortex for the first time, we uncover a U-shaped eccentricity gradient which reverses near the lateral fusiform. These findings produce a parsimonious model of cortical organization incorporating previously uncharacterized regions, offering a new organizing principle of high-level vision.

## Introduction

High-level visual cortex (HLVC), which spans the ventral and lateral surfaces of the human temporal lobe, comprises clusters of neurons involved in the perception and recognition of ecologically relevant stimuli such as faces, words, places, and limbs (Gross et al., 1972; Cohen et al., 2000; Pinsk et al., 2005; Downing et al., 2006). These category-selective regions are thought to emerge in an experience-dependent manner across childhood (Scherf et al., 2007; Golarai et al., 2015; McKyton et al., 2015; Arcaro et al., 2017; Thompson et al., 2017; Gomez et al., 2019a; Huber et al., 2021). The functional organization of these category-selective regions is strikingly consistent across individuals, whereby each region’s location is anchored to a particular cortical fold (Weiner et al., 2014; Grill Spector et al., 2017; Natu et al., 2021). In addition to being responsive to a preferred visual stimulus, each region receives information from preferred locations in the visual field (Kay et al., 2015; Silson et al., 2015; Le et al., 2017; Gomez et al., 2018). This region of visual space from which a neuron responds to a stimulus is the neuron’s receptive field (RF; Hubel and Wiesel, 1968). Because visual cortex is organized retinotopically, neighboring neurons have neighboring RFs. Neurons located more laterally within ventral temporal cortex (VTC) near the fusiform gyrus have central receptive fields responding to stimuli near the point of fixation, while those in the medial VTC have receptive fields which sample the periphery of the visual field more heavily (Levy et al., 2001). This results in a lateral-to-medial eccentricity gradient (Grill-Spector and Weiner, 2014) thought to partially emerge from feed-forward axonal connectivity (Wandell and Winawer, 2011; Kravitz et al., 2013) and is thus, to some extent, inherited from retinotopic visual field maps in earlier visual cortex. This interesting and surprisingly consistent pattern gave rise to the prominent eccentricity theory for functional organization in HLVC: objects viewed with central vision (e.g., faces) lead to specialized clusters in the cortex with central visual field input (e.g., lateral VTC), and objects experienced with peripheral vision lead to specialization in the cortex with input from the visual periphery (medial VTC; Hasson et al., 2002; Kay et al., 2015; Gomez et al., 2019a).

**Figure 1.**
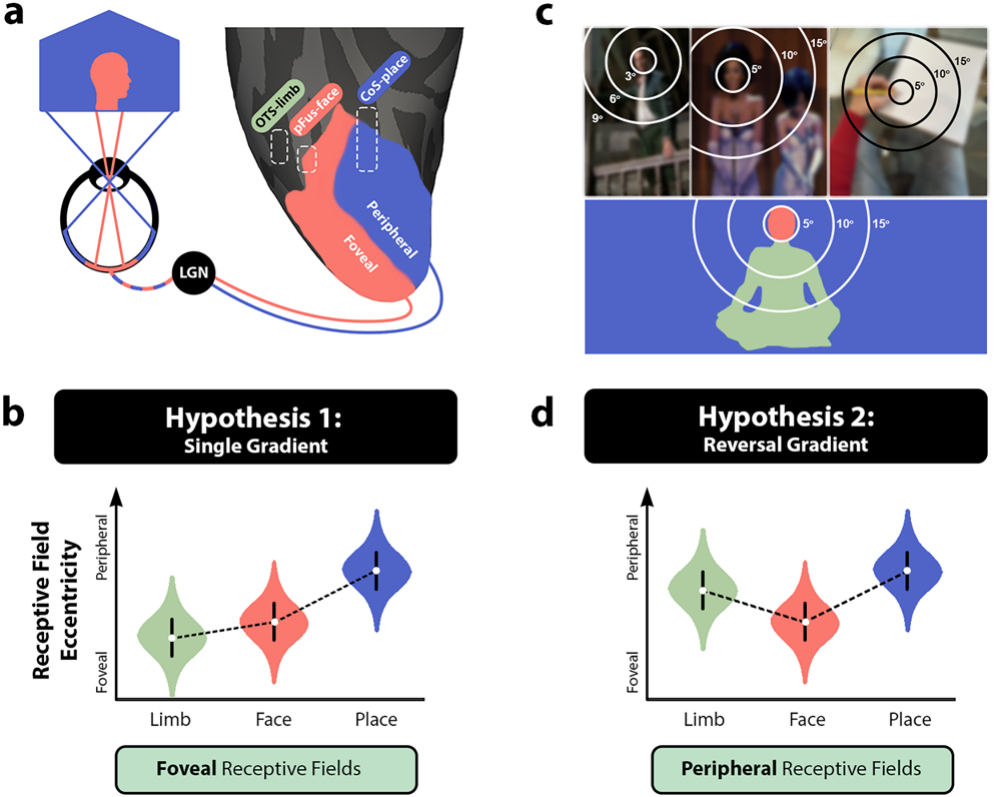
Predictions for limb-selective receptive field properties. ***a***, Illustration of retinal eccentricity within ventral temporal cortex (VTC). The pRFs that have been mapped to date show a trend in which lateral VTC generally samples the central visual field, while those located medially sample the visual periphery. The outline labeled pFus corresponds to the approximate location of a face-selective region on the posterior fusiform. The CoS outline is a place-selective region in the collateral sulcus. The occipitotemporal sulcus (OTS) outline denotes the limb-selective region of interest. ***b***, Hypothetical distributions of pRF eccentricity values (violin plots) in a model of VTC with a single medial-lateral eccentricity gradient, with lateral VTC showing a general preference for central visual space compared with the medial aspect. ***c***, Viewing behavior by humans in social contexts is heavily biased toward fixating on the face (top left images), positioning bodies and limbs more peripherally on the retina. Even when interacting with objects (top right image), one’s own limbs are more peripherally located in the lower visual field. This experiential model would predict that pRF properties would pool information from peripheral locations of the lower visual field. Images blurred to adhere to copyright policy. ***d***, Hypothetical distributions of pRF eccentricity in the experiential model of VTC in which the medial-lateral eccentricity gradient shows a reversal at or near face-selective cortex. Each category is colored according to the inset image of a sitting person.

Faces, for example, are best recognized with central vision (Hsiao and Cottrell, 2008; Poltoratski et al., 2021). Across development, face-selective regions emerge on the lateral posterior and middle fusiform gyrus (pFus and mFus) in VTC (Fig. 1*a*). Scenes often extend well into our peripheral vision, and a scene-selective region is concordantly located more medially within the collateral sulcus (CoS; Fig. 1*a*). Despite the fact that it abuts face-selective cortex, receptive field properties of a region involved in the perception and recognition of limbs (Peelen and Downing, 2005; Taylor et al., 2007; Weiner and Grill-Spector, 2013) have yet to be mapped. The location of the limb-selective region, nestled laterally within the occipitotemporal sulcus (OTS), is near face-selective regions previously described as having a bias for central visual space (Hasson et al., 2002; Kay et al., 2015). Given this proximity, and the idea that lateral VTC might have a general preference for central visual space, one could hypothesize that this limb-selective region will similarly show a preference for visual input from the center of visual space (Fig. 1*b*). Is this consistent with how limbs are behaviorally experienced as a visual stimulus?

In the vast majority of social interactions, humans fixate their gaze on the face (Yarbus, 2013; Amso et al., 2014), placing body and limb information more peripherally within the visual field. Even when alone, one’s own limbs during work or ambulation are usually located below the point of one’s fixation (Fig. 1*c*). Thus, an experiential account of limbs would predict peripherally located receptive fields within limb-selective cortex. When traveling medial to lateral in VTC, this would result in a reversal of the broader eccentricity gradient (Fig. 1*d*), a phenomenon not yet observed. We hypothesize that limb-selective cortex will show peripherally biased population receptive fields (pRFs) despite its lateral location bordering the foveally biased face-selective regions. While this prediction is consistent with the premise that a category-selective region’s spatial processing is consistent with how objects are experienced (Levy et al., 2001; Hasson et al., 2002), peripherally biased pRFs would contradict the idea that lateral VTC has a general preference for central visual space (Gomez et al., 2019a).

Differentiating these outcomes would require mapping both visual category representation and RF properties in lateral VTC, within the same individuals. However, most previous pRF mapping studies employed high-contrast checkerboard stimuli, a stimulus that is relatively inefficient at driving strong responses in high-level cortex where the limb-selective region is located (Finzi et al., 2021). Here we created a retinotopic mapping paradigm with rich social content capable of driving visual responses well beyond primary visual cortex. Combined with naturalistic eye-tracking data, we ask: Do pRFs of limb-selective cortex, like face-selective regions, show a foveal bias, resulting in a general preference for central visual space in lateral VTC (Fig. 1*a,b*), or do the spatial computations performed by limb-selective cortex align with the peripheral retinal location of limbs as they are experienced visually (Fig. 1*c,d*)? In answering these questions, we hope to provide a more parsimonious framework describing the functional organization of visual cortex that accounts for these seemingly disparate predictions.

## Materials and Methods

### Participants

A total of 27 participants aged 18–20 (mean ± SD 18.6 ± 0.75 year, 17 females) participated in the fMRI study. One additional participant could not remain awake during fMRI and was thus excluded from analyses. A total of 25 separate participants (age mean ± SD 23 ± 8.6 year, 13 females) participated in the eye-tracking experiment. Two additional participants were excluded from the eye-tracking dataset due to insufficient data collected due to technical difficulties. All participants were right-handed with normal or corrected-to-normal vision and provided informed, written consent to participate in the experiment. Procedures were approved by the Princeton Internal Review Board on human subjects research. Retinotopy data from the publicly available HCP 7T Retinotopy Dataset were collected from a total of 181 subjects (109 females), as described in the original publication (N. Benson et al., 2018).

### Data acquisition

MRI and fMRI data were collected at the Scully Center for the Neuroscience of Mind and Behavior within the Princeton Neuroscience Institute, using a Siemens 3 T Skyra system. Each participant completed a single recording session in which they completed four runs of a visual category localizer experiment (Stigliani et al., 2015; 4 m each), followed by a qMRI anatomical scan (Mezer et al., 2013, 2016; 25 m), a break outside the scanner (5 m), and three runs of the pRF mapping experiment (5 m each).

#### Quantitative magnetic resonance imaging acquisition

An artificial T1-weighted image was produced using the following quantitative magnetic resonance imaging (qMRI) protocol for the purposes of reconstructing the cortical surface. Quantitative MRI was used to estimate T1 relaxation times based on existing protocols (Mezer et al., 2013, 2016), measured from three spoiled gradient echo (spoiled-GE) images with flip angles of 4, 10, and 20°. An artificial T1-weighted anatomical 3D image was then produced from these measurements for each participant for the purposes of segmenting white and gray matter through FreeSurfer software.

#### Functional MRI

For the visual category localizer, fMRI scans comprised 72 slices acquired using a multiplexed echo planar imaging (EPI) sequence (multiband acceleration factor, 3; voxel size, 2.5 mm isotropic), with repetition time (TR), 2 s; echo time (TE), 30 ms; and flip angle (FA), 80°. Each run was 209 s in duration and there were four runs in total. For the retinotopic mapping experiment, scan parameters were identical with the exception of multiband acceleration factor set to 2 and 48 total slices.

#### Eye-tracking data

The behavioral experiment involving movie watching was conducted within the Scully Center for the Neuroscience of Mind and Behavior using an SR Research EyeLink 1000 Plus system. Visual stimuli were displayed on an Asus LCD monitor (PG278QR: 2,560 × 1,440p, 120 Hz refresh rate, ultralow motion blur enabled) and controlled with an Apple Mac Pro running OSX 10.11. The stimulus monitor measures 597 mm wide, located 570 mm from the viewer’s eyes, resulting in a retinal image of 55 by 33 degrees of visual angle. Participants’ eyes were calibrated using 5 point calibration (four corners, one center) and validated before movie viewing began. Eye gaze position was sampled and recorded at a rate of 1,000 Hz.

### Eye-tracking Hollywood movies experiment

**Figure 2.**
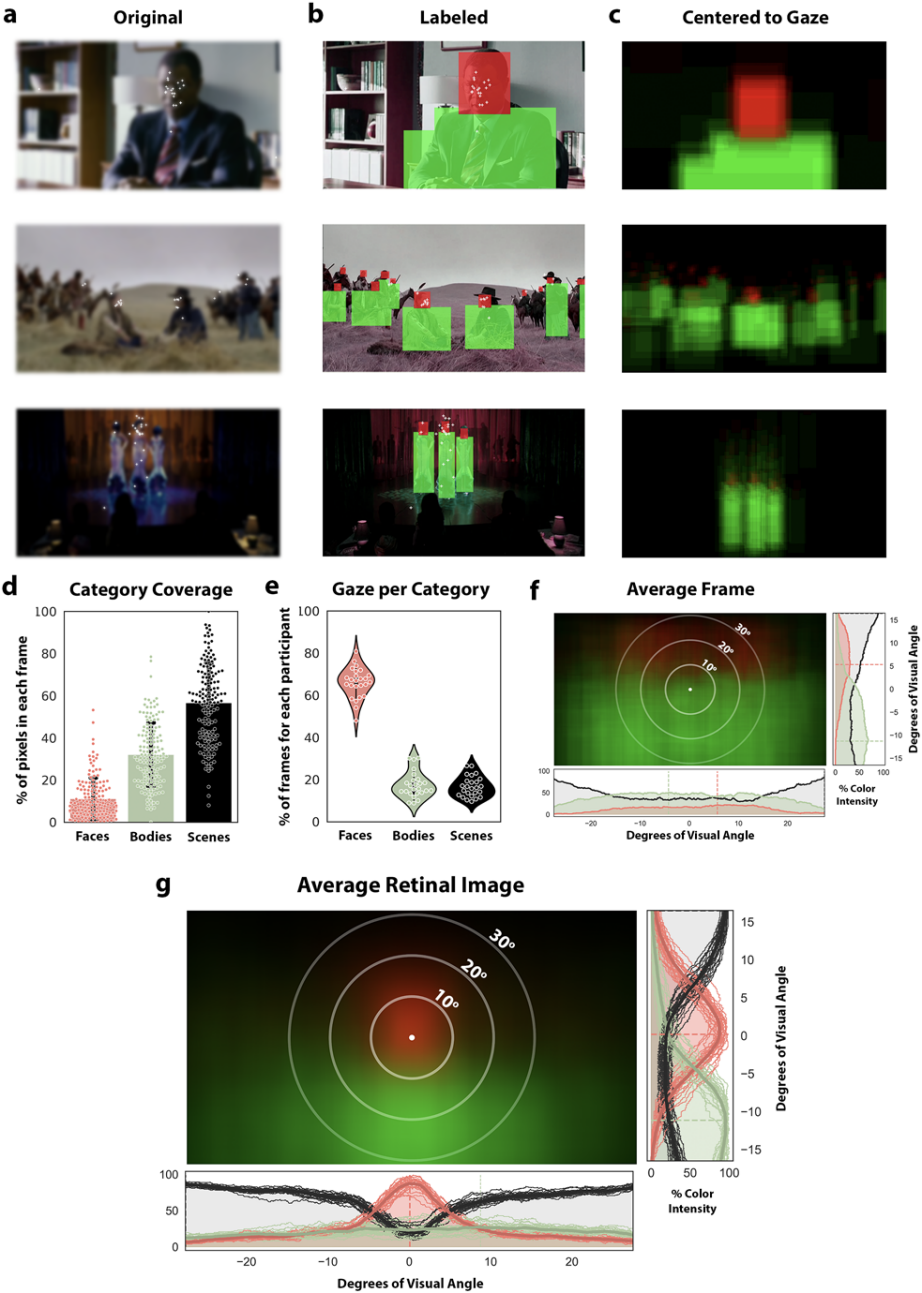
Quantification of viewing experience suggests bodies are mostly viewed in the lower periphery of the visual field. ***a***, Example frames from video clips included in the “Hollywood” dataset (Costela and Woods, 2019), with eye fixations of *N* = 25 participants overlaid as white plus signs. Images blurred in figure to adhere to copyright policy. ***b***, Frames were manually labeled with “face” and “body” labels. ***c***, Average “retinal image” from a given frame translated to center the point of fixation, averaged across participants. ***d***, Quantification of how much of the visual field was occupied by faces (red), bodies (green), or other objects and scenes (black) from extracted movie frames. Each dot is a frame, with a total of 150 frames. ***e***, Violin plots demonstrating the distribution of viewing statistics across all participants and frames. Each dot represents data from a single participant and visual category, across all frames. ***f***, The average image across all extracted frames, without aligning to fixation. Bodies (green) statistically occupy the center of the video screen, faces (red) the upper visual field, and scenes (black) the visual periphery. Distribution plots demonstrate color/category observation probability at each point along the x/y axis. Dashed lines indicate distribution peak. ***g***, The average retinal image across all extracted frames and all participants centered according to participant fixation. Increasing intensity of red or green signifies increased probability of a face or body, respectively, populating that point on the retina. Faces statistically occupy the center of the retina, bodies occupy the lower peripheral visual field, and scenes occupy the remaining visual periphery. Distribution plots demonstrate, for each point along either the x or y axis, the color/category observation probability across frames (normaized to the maximum). Thin distribution lines represent individual participants, thick lines show group mean distribution, and dashed lines mark distribution peaks.

Participants (*n* = 25) viewed 25 video clips of 30 s duration, taken from different Hollywood films (Costela and Woods, 2019). Movie clips were chosen such that they included people (with visible faces and bodies) either alone or engaging in social interactions, with other objects and a background scene present (Fig. 2*a*) to allow participants the option to fixate on a range of animate and inanimate stimuli in any given clip. Participants were instructed to watch movies as they normally would when enjoying a movie. Participant head motion was controlled using a table-mounted chin rest. To efficiently analyze data, a subset of frames were extracted from each movie clip at a rate of 0.2 Hz, resulting in six frame samples per clip, for a total of 150 frames. The same frames were extracted across participants to enable the quantification of the consistency of the retinal image produced during a given scene. Face and body stimuli were hand labeled using rectangles that subtended the vertical and horizontal extent of the pixels occupied by the face or body, where faces were assigned RGB values of [255,0,0] and bodies were assigned RGB values of [0,255,0]. All other pixels were defined as black [0,0,0] such that each pixel of each frame was categorized as face (red), body (green), or scenes (black). To produce a simulation of the retinal image within a participant according to their point of fixation at a given frame, the extracted frame was padded with RGB values of zeros, the image was centered/translated such that the point of fixation was brought into the center of the frame, and then the image was cropped back to the dimensions of the viewing monitor.

### Visual category localizer

**Figure 3.**
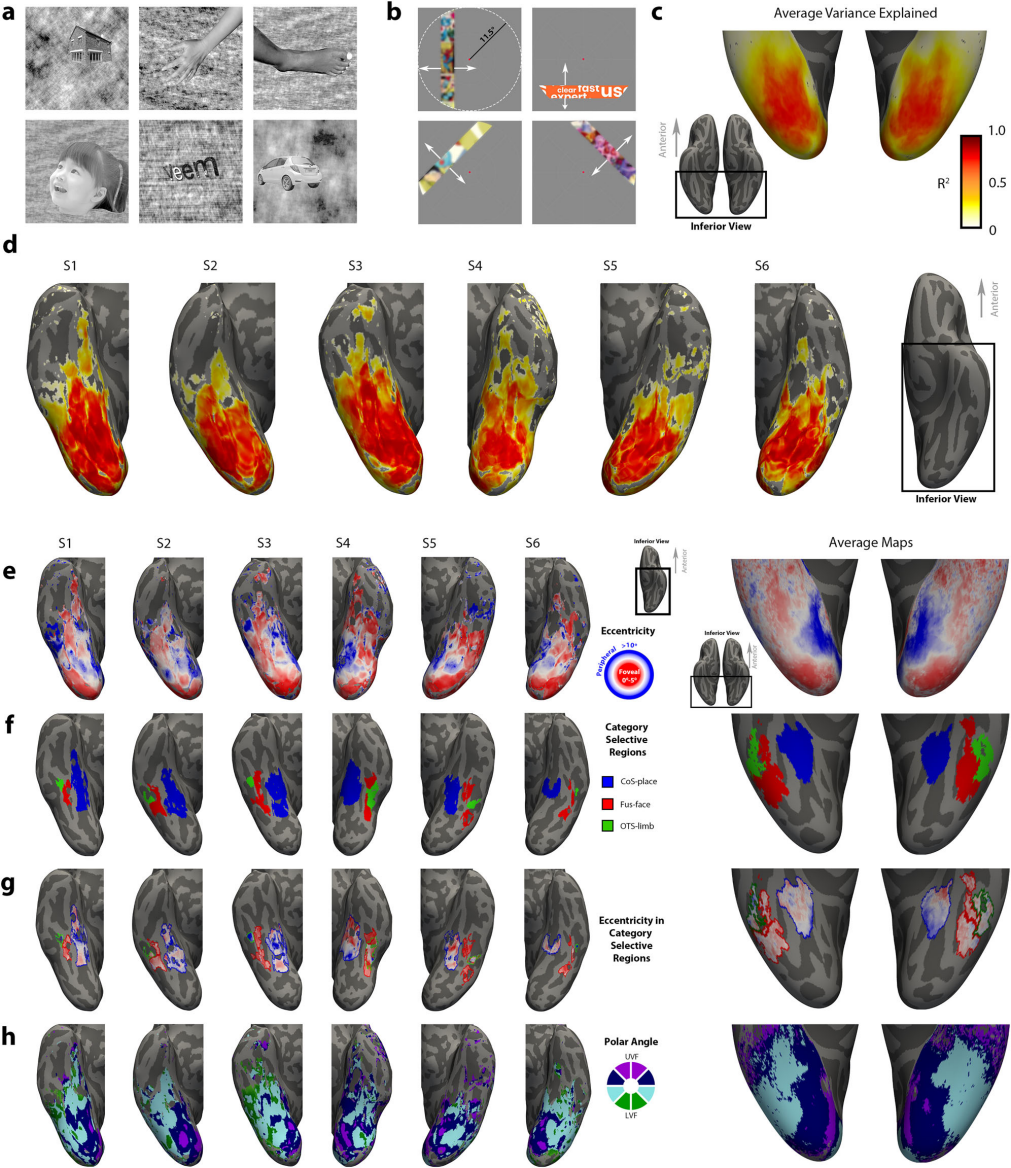
fMRI experimental design, example participants, and average maps. ***a***, A visual category localizer (Stigliani et al., 2015) allowed us to localize category-selective regions in each individual brain. Images belonged to six visual categories: houses, hands, feet, faces (adult + child), pseudowords, and cars. ***b***, Illustration of the population receptive field (pRF) mapping experiment in which a sweeping bar containing flickering cartoon images swept across the screen on the vertical, horizontal, and diagonal axes. Fixation point enlarged for visibility. White arrows indicate directions of motion. Images blurred in figure to adhere to copyright policy. ***c–h***, Example data and group averages in an inferior view of ventral temporal cortex (VTC). Individual maps show six inflated hemispheres from six adults, displayed in their native cortical surface (S1-S6). Average maps (from *n* = 27 participants) displayed on an inflated cortical surface of the FreeSurfer average brain. Inset brains show zoomed regions in black outline. ***c***, Average variance explained map derived from the pRF mapping experiment. Each vertex is colored according to the fit pRF’s variance explained (*R*^2^), where darker colors indicate higher variance explained. ***d***, Variance explained maps from six example hemispheres (*N* = 6 adults) in native brain space. Note that VTC is covered at >10% variance explained. ***e***, Eccentricity maps derived from pRF mapping. Each vertex is colored according to the eccentricity value of its pRF model fit. Warmer colors indicate lower eccentricity (more foveal) while cooler colors represent preference for more eccentric (peripheral) stimuli. Individual data thresholded by variance explained greater than 10%, average map unthresholded. ***f***, Labeling of limb- (green), face- (red), and place-selective (blue) regions in ventral temporal cortex (VTC) based on the contrasts derived from the visual category localizer experiment (*T*-values >3) shown on individual brains or as probabilistic averages on the average cortical surface (Rosenke et al., 2021). ***g***, Eccentricity in VTC category-selective regions—intersection of panels ***e–f***. ***h***, Polar angle maps. Individual data thresholded by variance explained greater than 10%, average map unthresholded.

During each run (209 s), participants fixated on a central dot as visual stimuli were presented in 4 s blocks. Each block consisted of eight images alternating at 2 Hz, from a single visual category out of six in total: houses, hands, feet, faces (adult + child), pseudowords, and cars (Fig. 3*a*). Limb stimuli included both upper and lower limbs and always included the digits. We avoid using body stimuli for two reasons: (1) we want to maximize the probability we are measuring neural populations which represent limbs and (2) minimize activity from neighboring face-selective neurons which have been shown to respond to the implied presence of a face or head when using headless body stimuli (Arcaro et al., 2020). Each image was tightly cropped and overlaid on a textured grayscale background produced by phase scrambling a stimulus from another category, where an additional baseline condition consisted of a scrambled background with no overlaid image (Stigliani et al., 2015). Images subtended a visual angle of 7.125° centered on the fovea and were presented with PsychoPy (Peirce et al., 2019). To ensure attention to the stimuli, participants completed a two-back task, where they were instructed to press a button whenever they detected that an image repeated itself with exactly one intervening image between repeats.

### Population receptive field mapping

During each run (300 s duration), participants fixated on a central dot as a bar (2° visual angle) swept across the screen on the vertical, horizontal, and diagonal axes with two directions of motion, revealing portions of a large circle defining the full visual field (radius, 11.5°; 8 bar sweeping configurations; Fig. 3*c*). The bar displayed a strip of colorful images from a selection of >100 colorful cartoons and comics, alternating at a rate of 7 Hz. This experiment is referred to as “Cartoonotopy.” At regular intervals, the apertures were removed and participants viewed a mean luminance gray background with a central fixation point. The cartoon stimuli were chosen to represent a wide array of stimuli including bodies, faces, words, houses, and objects. This experiment was adapted from our previous work (Finzi et al., 2021), with changes to the stimulus content (more evenly sampling different categories), image refresh rate (7 Hz rather than 8 Hz), and bar motion (smooth, continuous sweeping motion rather than discrete 2° steps). Another important difference from the previous version of Cartoonotopy is that participants attended to the visual stimulus (e.g., the sweeping bar) and monitored it for the appearance of wiggling bumble bees. Sweeping bars appeared on a low-contrast, radial grid resembling a spider web, and participants were instructed to detect the bees to help Charlotte the Spider remove them from her web. This narrative was designed specifically for a separate pediatric experiment but was also given to the present adult participants for consistency.

### Anatomical data analysis

qMRI data were processed using the mrQ software package in MATLAB to produce T1-weighted maps (Mezer et al., 2013). The full analysis pipeline and its published description is freely accessible at https://github.com/mezera/mrQ. The qMRI-derived artificial T1-weighted anatomical image was produced for each individual participant, segmented with FreeSurfer (https://surfer.nmr.mgh.harvard.edu/), and then passed through iBeat for an optimal separation of gray and white matter (Li et al., 2014, 2015) and fed back to FreeSurfer to correct the white matter segmentation. The resulting segmentations were used for cortical surface reconstruction (Dale et al., 1999; Fischl, 2012) and visualization of retinotopic data in each individual’s native brain space.

### Functional data analysis

Analyses were performed in each individual's native brain space. An inflated cortical surface reconstruction was used for each participant, and all functional data (localizer and pRF data) were projected onto this cortical surface in which voxel time-series data were resampled to each cortical vertex. Data was preprocessed based on the HCP minimal preprocessing pipeline for motion correction, slice-timing correction, and lastly phase-distortion correction using Topup (Andersson et al., 2003; S. M. Smith et al., 2004). No spatial smoothing was used. Resampling voxel time-series data to cortical vertices and general linear model (GLM) analyses were processed through FsFast (FreeSurfer Functional Analysis Stream; https://surfer.nmr.mgh.harvard.edu/) to derive for each voxel the beta-weights associated with each category for the production of contrast maps, described below.

### Labeling of category-selective regions

Category-selective regions of interest (ROIs) were defined for each individual participant using data from the visual category localizer experiment. For labeling each category-selective region, statistical contrasts of the category of interest > all other stimuli were thresholded at *T*-values>3 and overlaid onto the cortical surface for each subject. The borders around each of the category-selective regions were drawn on the cortical surface, and the vertices within the border above a threshold of 3 were defined as that region’s ROI for that participant.

For limb-selective regions, we used the statistical contrast of [hands > nonlimb stimuli]. The borders for each limb-selective ROI were determined using anatomical landmarks as detailed by Weiner and Grill-Spector (2011) and included limb-selective regions on the occipitotemporal sulcus (OTS-limb), lateral occipital sulcus (LOS-limb), inferotemporal gyrus (ITG-limb), and middle temporal gyrus (MTG-limb). For localization of face-selective regions, we used the contrast [face > all other stimuli] and included face-selective regions on the inferior occipital gyrus (IOG-face), the posterior fusiform (pFus-face), the middle fusiform gyrus (mFus-face), and the posterior branch of the superior temporal sulcus (pSTS-face). For localization of the place-selective region, we used the contrast [house > all other stimuli] and included a single place-selective region on the collateral sulcus (CoS-place). All labeled regions are shown on an example right hemisphere of a single participant in Figure 5*a*.

**Figure 4.**
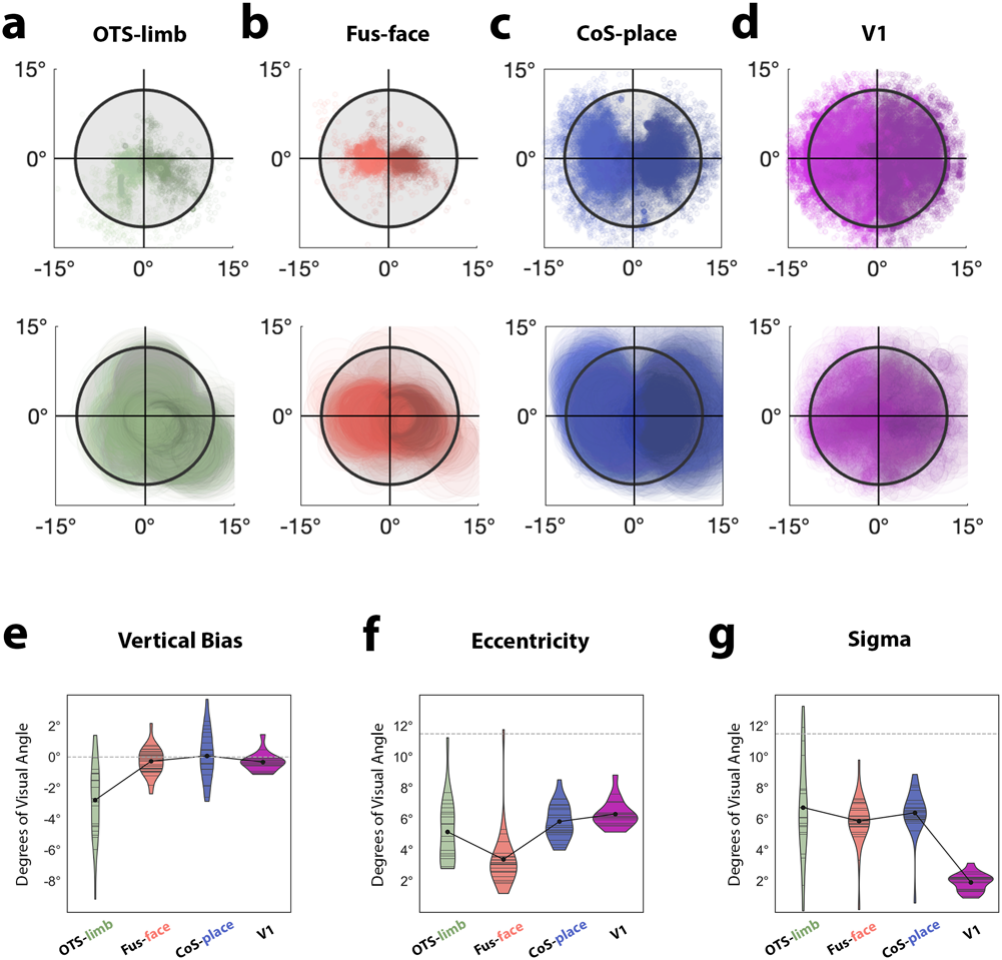
Combined category localization and population receptive field (pRF) mapping supports an experiential account of high-level visual cortex organization. ***a–d***, Scatterplots of pRF centers (the *x* and *y* model fit parameters) in each cortical region (top row), and raw pRFs (center + size; bottom row). Each dot represents the spatial location of a pRF center from a single vertex from a single participant overlaid such that darker areas indicate locations of higher overlap. *x*- and *y*-axes show degrees of visual angle. Black circle denotes stimulus presentation area (11.5° radius). OTS-limb, limb-selective region in the occipitotemporal sulcus. Fus-face, face-selective regions on the posterior fusiform gyrus. CoS-place, place-selective region in the collateral sulcus. V1, primary visual cortex. ***e–g***, Quantification of the pRF properties across regions in individual participants: ***e***, mean *y*-coordinate of pRF centers, ***f***, mean pRF eccentricity, and ***g***, mean pRF size, sigma. Violin plots demonstrate distribution of values over participants within each visual category. Horizontal lines indicate data from each individual, black dots represent group means, black lines connect group means.

### pRF modeling

Using the fMRI data acquired from the sweeping bar task, we performed pRF mapping (Tootell et al., 1997; A. T. Smith et al., 2001; Dumoulin and Wandell, 2008; Kay et al., 2013; Wandell et al., 2015; Wandell and Winawer, 2015), a computational modeling technique that allows quantification of pRF properties in each voxel. Sweeping the stimulus across the visual field while the participant maintains central fixation allows us to estimate what subset of the visual field each voxel is most responsive to. A receptive field is modeled for every voxel independently as a 2D Gaussian with a center (**x,y**) and a size determined by **σ**, the standard deviation term in the Gaussian model. Through an iterative process optimizing the Gaussian properties, the predicted and observed fMRI time-series are compared to find the pRF parameters that best predict the observed time-series for that voxel by minimizing the root mean square error. Software repository for pRF modeling was developed by the Stanford VISTA Lab (github.com/vistalab; Dumoulin and Wandell, 2008), with additions for compressive nonlinearity implemented by Kendrick Kay (Kay et al., 2013), as well as adaptations by the NYU Winawer Lab for use in FreeSurfer-style data formats (github.com/WinawerLab/prfVista; Himmelberg et al., 2023).

In regard to visualizing pRF data, receptive field centers and sizes can be visualized for each participant in each ROI based on the modeled pRFs using custom-made plots. Coverage plots are commonly used to compare the average pRF between ROIs, but averaging pRFs can result in a spurious coverage that does not reflect the actual properties of the underlying pRFs. Thus, aiming to minimize the use of mass averaging methods while presenting raw data whenever possible, in Figure 3*a-d* (bottom row), we plot an overlay of all the raw pRFs (*x*, *y*, *σ*) from all subjects on a single plot, such that darker regions indicate regions of significant overlap. The limitation of this visualization is that individuals that have more data points have more influence on the resulting image as the number of modeled vertices across participants is not constant, but no participant can contribute >500 vertices in this approach to minimize visualization bias. All statistical quantifications were random-effects analyses in which pRF properties from a given individual were averaged before entering statistical models. Finally, in Figure 3*a-d* (top row), we plot only the centers of pRFs to emphasize the spread of the pRFs across the visual field in the different visual regions.

### Replication with Human Connectome Project (HCP) data

To test whether our findings replicate in an independent dataset, we used the publicly available HCP 7T Retinotopy Dataset (N. Benson et al., 2018). Data were acquired from https://osf.io/bw9ec/wiki/home. Surface maps of vertex-wise pRF fits were transformed to fsaverage space, where we ran the same analysis pipeline on both datasets (Fig. 6). Differences in stimulus properties, as well as a comparison of variance explained and eccentricity values between the datasets, are shown in Figure 6*b–g*.

### Statistical analysis

Receptive field data were fit using a stimulus-encoding model (Dumoulin and Wandell, 2008) to derive parameters describing the circular Gaussian model fit for each vertex on the cortical surface. No spatial smoothing was performed on functional MRI data. In the fMRI analysis, the data used to define the ROIs were independent of the data used to calculate the pRF properties (visual category localizer task and pRF mapping task, respectively), to avoid circularity confounds. When comparing pRF parameters between ROIs, within-subjects statistics were used, such as paired *t* tests and within-subjects ANVOAs. Statistical analyses comparing pRFs were done using random-effects models, with each participant contributing a single data point summarizing a given pRF property from a cortical region. Where possible, all individual data points were shown directly (e.g., swarm plots) or shown within visual summary illustrations (e.g., violin plots). Data visualizations were made using custom-built scripts in MATLAB (The MathWorks), as well as using Matplotlib (Hunter, 2007) and Seaborn (Waskom, 2021) Python packages. Resulting *p* values quantifying statistical significance were always two tailed. The degrees of freedom for each analysis (or number of participants within an analysis) are reported following the relevant statistic within the text. Data were assumed to be normal, with no gross violations of normality observed. Given that data points are compared in a within-subjects manner with no grouping of participants, group randomization was not relevant in this particular study. No statistical methods were used to predetermine sample sizes, but our sample sizes are similar to those reported in previous publications using HCP data, as well as in previous work (Gomez et al., 2019b).

## Results

### What are the retinal statistics of limbs as they are visually experienced?

A wide body of research posits that visual experiences in childhood have influential effects on cortical development and functional organization in adulthood (Scherf et al., 2007; Srihasam et al., 2014; Golarai et al., 2015; Arcaro et al., 2017). In fact, biases in the way different stimuli are viewed during development are thought to lead to the distinct and consistent organization of category-selective regions in HLVC (Levy et al., 2001; Hasson et al., 2002; Malach et al., 2002; Gomez et al., 2018, 2019a). Faces are predominantly viewed with central vision across development (Gomez et al., 2018), yet how limbs and bodies are visually experienced during naturalistic viewing is less clear. While bodies and limbs have been shown to have lower saliency than faces (Broda and de Haas, 2022), their statistical location on the retina compared with other categories like faces or scenes has not been quantified. Given the prominence of faces in social interactions, and the tendency to fixate on faces, we hypothesize that limbs and bodies will consistently result in more peripheral retinal images, particularly in the lower visual field (below fixation on faces).

To test this hypothesis, a total of 25 individuals (average age 23 ± 8.6 years, 52% female) participated in a naturalistic eye-tracking study. Each individual viewed 25 video clips, each 30 s long, taken from different Hollywood films (Costela and Woods, 2019). Movie clips were chosen such that they included people (with visible faces and bodies) either alone or engaging in social interactions, with other objects and a background scene present (Fig. 2*a*). Background scenes included furnished rooms, outdoor scenes, and large rooms (Fig. 2*a*), all of which have been shown to drive activity in place-selective cortex (Epstein and Kanwisher, 1998; Stigliani et al., 2015). Participants were instructed to watch the clips as they normally would when enjoying a movie with the exception that head motion was controlled through a chin rest, and fixations were eye-tracked for the duration of the experiment. To assess the retinal image that bodies and limbs produce, a subset of frames were extracted from each movie clip, sampled at 0.2 Hz (*n* = 6 frames per clip, total of *n* = 150 frames). Example fixations on selected frames are illustrated in Figure 2*a*. Each extracted frame was hand labeled to produce a binarized mask denoting the location of faces and bodies present within each frame (Fig. 2*b*). Using these binary masks, we simulated the average retinal image during viewing by shifting each frame so that the point of fixation became the new image’s center (Materials and Methods). We averaged the fixation-centered images across participants for each frame, such that regions with brighter colors indicate more consistency across participants (Fig. 2*c*).

To quantify these visual statistics, we performed two analyses. First, we quantified what proportion of looking time was spent on each visual category by extracting identical moments (i.e., frames) of each movie (*n* = 6 frames per clip). For each participant, we measured the fraction of extracted frames on which a face, a body/limb, or background/scene was being fixated (Fig. 2*e*). We find that faces are the locus of fixation in 66% of the 150 frames (on average over participants), whereas bodies and scenes were fixated on in 17.4% and 16.6% of frames, respectively. That is, we find that faces were more than three times more likely to be the focus of participant fixation compared with either bodies (*T*_[26]_ = 19.6; *p* < 0.001; CI = [43.42, 53.66]; Fig. 2*e*) or to scenes (*T*_[26]_ = 23.2; *p* < 0.001; CI = [44.99, 53.79]). One possible confound that is important to address is whether this dramatic difference in gaze location could be explained by the coverage of the frames that each category occupies. That is, could it be that faces are shown more frequently or in larger size than bodies, driving the difference in gaze time between those visual categories? In Figure 2*d* we quantified the percent of pixels in each frame devoted to each visual category. On average, faces occupied 11% of pixels, bodies 32%, and scenes 57% of the pixels. That is, even though bodies and limbs occupy three times the pixels that faces do on average (*T*_[149]_ = 15.0; *p* < 0.001; CI = [0.18 0.24]; Fig. 2*d*), faces are still fixated a factor of three times more often (Fig. 2*e*).

Second, we sought to quantify the average retinal image produced during movie watching. To produce a model of the inherent visual statistics of the movie frames themselves, we averaged all the labeled video frames. In the average image, consistent patterns across frames should result in regions of solid bright colors, and if there are no consistent patterns across frames, the average image should be completely black or very blurred, with flat color/category intensity distributions. In the average image (Fig. 2*f*), we observe that bodies tend to be shown in the bottom two-thirds of the screen, while faces are more often shown on the top third of the screen, with both categories demonstrating a smooth distribution across the extent of the *x*-axis. Importantly, we see that the innermost circle in the center of the screen (Fig. 2*f*) is entirely green, indicating that bodies most consistently occupy the center of the video frame, more than other categories. Thus, a viewer who was biased toward always fixating near the middle of the screen would be foveating most frequently on body stimuli. We next sought to quantify the average retinal image by taking into account fixation data. Each extracted video frame was padded and translated so that the point of fixation was centered (Fig. 2*c*), and these fixation-centered frames were averaged across all movie clips and participants (Fig. 2*g*). As illustrated in the histograms quantifying the proportion of stimulus category density as a function of *x*- or *y*-axis position (Fig. 2*g*), faces consistently occupy what would be the fovea, indicated by the bright red region surrounding the point of fixation. Bodies, conversely, were consistently observed outside the point of fixation and did not begin surpassing faces in their retinal image frequency until about 10° below the point of fixation.

When quantifying the differences in stimulus intensity along the vertical meridian between the face (red) and body (green) distributions (Fig. 2*g*, rightmost inset), we find that the average *y*-coordinate for peak stimulus intensity of faces was significantly higher in the visual field compared with that of bodies (mean *y*-coordinate of peak intensity for faces = 0.17 ± 1.06°, bodies = −11.6 ± 1.47°; paired *T*_[24]_ = 50.78; *p* < 0.001). Quantifying differences in the *x*-coordinate of peak stimulus intensity along the horizontal meridian (Fig. 2*g*, bottom inset), we find a smaller difference between face and body stimuli, where the between-subject variability in peak intensity of body stimulus was much higher than in face stimuli (mean *x*-coordinate of peak intensity for faces = 0.08 ± 0.66°, bodies = 8.76 ± 8.42°; paired *T*_[24]_ = −3.74; *p* = 0.001). It is worth noting this variability is likely driven by the largely even distribution along the horizontal axis, in which even small biases in fixation will result in a largely shifted peak. Had faces and bodies been fixated on equally during movie watching, we would have seen a region surrounding the fovea showing no preference for either category. However, with faces commanding a large majority of the viewers’ visual attention (Fig. 2*e*), bodies and limbs, given their physical position below the head, statistically fall beneath the point of fixation, matching well the average retinal image shown in Figure 2*g*. Taken together, these findings suggest that consistently across individuals and visual scenes, faces tend to be fixated on, while limbs tend to lie in the lower periphery of the retinal image.

### Do limb-selective population receptive fields show a foveal or peripheral bias?

To quantify the pRF properties of limb-selective cortex, we recruited *n* = 27 undergraduate students (17 females, aged 18.6 ± 0.75) to undergo functional MRI while completing two perceptual experiments. The first was a visual category localizer presenting images of various categories (faces, limbs, words, houses, objects) while participants underwent functional magnetic resonance imaging (Fig. 3*a*). Localizer data were used to identify limb-selective voxels using the contrast hands > all other nonlimb stimuli, thresholded at *T*-values >3 (Materials and Methods). We avoid full-body stimuli to minimize costimulation of face-selective regions (Arcaro et al., 2020; Materials and Methods). In each participant, we defined the limb-selective region located within the occipitotemporal sulcus (OTS), at a position approximately half of the sulcus’ length (Fig. 3*f*). We will refer to this location as OTS-limb (Weiner and Grill-Spector, 2013), sometimes referred to in the literature as the extrastriate or fusiform body area (EBA or FBA; Downing et al., 2001; Peelen and Downing, 2007; Taylor et al., 2007). Example OTS-limb regions are illustrated in six participants in Figure 3*f*. To quantify OTS-limb pRF properties relative to its face- and place-selective counterparts, we identified in every participant a face-selective region located on the posterior fusiform gyrus, which we refer to as pFus-face and mFus-face (Weiner and Grill-Spector, 2013), sometimes collectively referred to as the fusiform face area (FFA; Puce et al., 1995; Kanwisher et al., 1997). We also identified the place-selective region (houses > all other stimuli; *T*-values >3) located more medially in the collateral sulcus (CoS), referred to here as CoS-place, and sometimes in the literature as the parahippocampal place area (PPA; Aguirre et al., 1998; Epstein and Kanwisher, 1998).

To measure the pRF properties of category-selective voxels, the same participants underwent a retinotopic mapping experiment during fMRI, adapted from our previous work (Finzi et al., 2021). In a given run, participants fixated on a central dot while a sweeping bar moved across the screen, in which colorful cartoon images flickered at a rate of 7 Hz (Fig. 3*b*), referred to as “Cartoonotopy.” For each voxel, the population receptive field was modeled as a two-dimensional Gaussian encompassing the area of the visual field that elicited a reliable response from the voxel (Dumoulin and Wandell, 2008). The pRF is a stimulus-encoding model, yielding interpretable variables such as the location of the Gaussian center (*x*, *y*), its size (*σ*), and its gain (*g*). We also fit an exponential term to describe the nonlinearity of the response that occurs in voxels beyond primary visual cortex (V1; Kay et al., 2013, 2015). In example participants (Fig. 3*e*), each vertex of the cortical surface is colored according to the pRF’s radial distance from the point of fixation (i.e., eccentricity). With vertex-wise data thresholded at 10% variance explained, reliable pRFs can be modeled well beyond earlier visual cortex. The mean variance explained across pRFs within category-selective regions is quite high across participants (OTS-limb, 33 ± 13%; Fus-face, 59 ± 15%; Cos-place, 50 ± 11%; V1, 48 ± 7%). Because the localizer and pRF mapping data are aligned to each individual’s native cortical surface, we can ask how pRF properties differ between category-selective regions.

We find that pRFs in the OTS-limb region (Fig. 4*a*) are significantly more eccentric and demonstrate a unique lower visual field sampling bias when compared with Fus-face (Fig. 4*b*). Qualitatively, when pRFs are plotted on the visual field, the OTS-limb region has a denser coverage of the lower visual field compared with Fus-face, whose pRFs are more centrally biased, huddling around the point of fixation. In contrast, pRFs of the CoS-places region (Fig. 4*c*) have centers scattered above and below the horizontal meridian, with coverage extending well into the periphery. As a comparison, the receptive fields from V1 are plotted as well, with pRFs more evenly tiling the visual field (Fig. 4*d*). We quantified differences in receptive field properties (size and eccentricity) of the different category-selective regions, including only vertices that had variance explained of *R*^2^ > 0.1.

First comparing the *y*-coordinate of the pRF fits, we find that pRFs of the OTS-limb region have a stronger verticality bias (Fig. 4*e*), where the average *y*-coordinate is located significantly lower in the visual field (2.58° below the point of fixation) compared with Fus-face (1.33° below; *T*_[17]_ = −2.2; *p* = 0.042; CI = [−2.03,−0.04]) and CoS-place (0.83° below; *T*_[17]_ = −3.36; *p* = 0.004; CI = [−2.64,−0.60]). We further quantified this bias, asking what fraction of pRFs are located in the lower versus the upper visual field. In each participant, we found the percentage of pRFs whose center was in the lower visual field and compared the percentages in face and limb regions using a paired-samples *t* test. While all three category-selective regions showed a preference for the lower visual field, we found that in OTS-limbs, the percent of centers in the lower visual field was significantly higher than in CoS-place (Fig. 4*e*, limb, 70.09%; place, 57.31%; *T*_[17]_ = 2.53; *p* = 0.021) and numerically higher than Fus-face, although this difference was not statistically significant (Fig. 4*d*, limb, 70.09%; face, 60.84%; *p* = 0.176).

Comparing pRF properties across visual categories, we found that pRFs in OTS-limb were significantly more eccentric than in the Fus-face region (Fig. 4*f*, limb, 5.8°; face, 3.6°; *T*_[13]_ = 3.36; *p* = 0.005; CI = [1.01,4.89]). We found no significant difference in the size of the pRFs across those visual regions (Fig. 4*g*, limb, 7.01°; face, 6.49°; *T*_[13]_ = 1.01; *p* = 0.33; CI = [−0.89, 2.44]). In sum, combining pRF mapping with a visual category fMRI localizer, we demonstrate that limb-selective cortex in HLVC contains relatively peripheral receptive fields biased toward sampling the lower visual field when compared with face-selective regions.

### Is peripheral bias a general feature of both limb-selectivity as well as lateral temporal cortex?

**Figure 5.**
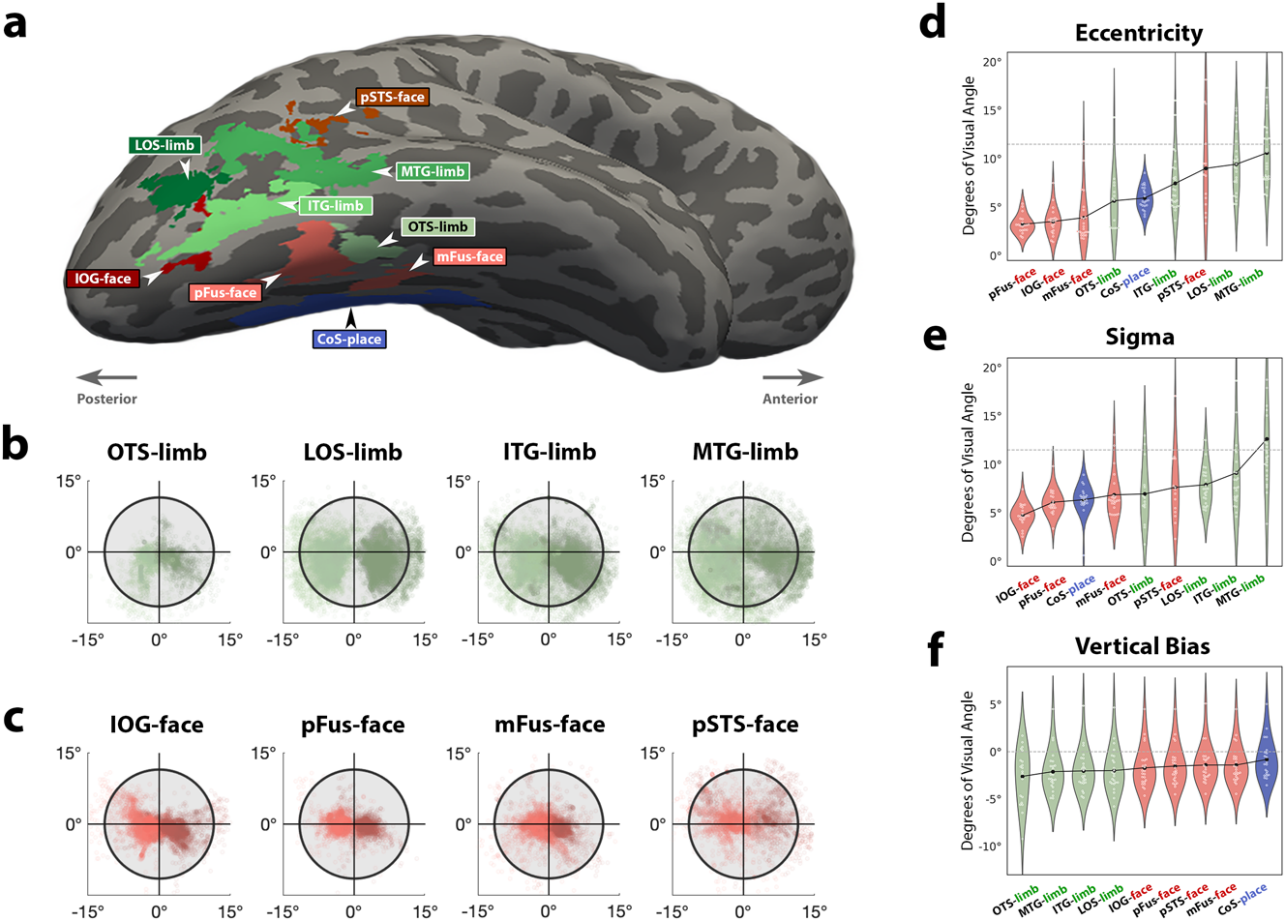
Receptive field properties across lateral and ventral high-level visual cortex. ***a***, Right hemisphere of a single participant showing the spatial distribution of the category-selective regions on this participant’s native cortical space. Labels drawn based on results from visual localizer using anatomical landmarks for accurate labeling in native brain space. ***b***, ***c***, Scatterplots depicting locations of pRF centers in each ROI in the limb ***b***, and face ***c***, selective regions. *x*- and *y*-axes are in degrees of visual field. Dot, the spatial location of a pRF center from a single vertex from a single participant. Dots are semitransparent to visualize pRF center overlap. Within each plot, darker colors indicate data from the left hemisphere, light colors from the right. Black circle marks stimulus borders at 11.5°. ***d–f***, Violin plots depicting pRF eccentricity (***d***), size (***e***), and vertical bias (***f***) across ROIs in the HLVC. White circles indicate individual data, black dots mark group mean in each ROI, and black lines connect group means. *x*-axis order ranked by value on *y*-axis, showing the regions with lowest average eccentricity (***d***), size (sigma) (***e***), or vertical bias (y-value) (***f***) on the left and regions with the highest values on the right. ***f***, Vertical bias in each ROI quantified as the average *y*-value of pRF centers in each participant.

If peripheral pRFs result from visual experience with limbs, then a limb-selective region should show a peripheral and lower visual field bias regardless of its cortical location. In the lateral visual stream (Pitcher and Ungerleider, 2021), there are three additional limb-selective regions which surround motion-selective cortex (area hMT+) in the ascending portion of the inferior temporal sulcus (Dumoulin et al., 2000). To test if these three regions also show a peripheral and lower visual field bias, we defined in the same participants the additional limb-selective regions (Weiner and Grill-Spector, 2011) located on the inferior temporal gyrus (ITG-limb), the lateral occipital sulcus (LOS-limb), and the middle temporal gyrus (MTG-limb). The spatial distribution of those regions is shown in an example participant in Figure 5*a*. Extracting the pRFs from each region’s voxels using the same pRF modeling protocol, we can plot the fit receptive field centers on the visual field (Fig. 5*b*). It is readily visible that all three lateral limb-selective regions have, like the ventral OTS-limb, pRF centers that show a bias toward sampling the lower visual field but extend even further into the periphery. Extending this analysis to additional lateral face-selective regions on the inferior occipital gyrus (IOG-face) and the posterior superior temporal sulcus (pSTS-face), we find that pRF centers on these more lateral face-selective regions are also more peripherally biased than their ventral face-selective counterparts (Fig. 5*c*).

Indeed, ranking these category-selective regions by their mean pRF eccentricity reveals that the lateral pSTS-face is more peripherally biased than ventral face regions and that all of the lateral limb-selective regions, together with pSTS-face, are the most peripherally biased (Fig. 5*d*). While consistent with previous observations of pSTS-face being less foveally biased than ventral regions (Pitcher et al., 2020; Finzi et al., 2021), the observation that pSTS-face more heavily samples the visual periphery, when viewed in the context of the peripheral bias of lateral limb-selective regions, suggests that peripheral visual field coverage may be a general feature of lateral visual cortex organization. In fact, this effect is so extreme that all the lateral limb-selective regions and pSTS-face show a larger bias toward the peripheral visual field than even the CoS-place region (Fig. 5*d*), a region hallmarked by its peripheral visual bias. When ranked by mean pRF size, we see a categorical split: ventral face-selective regions cluster together with the smallest pRF sizes, compared with lateral limb-selective regions which show the largest (Fig. 5*e*). Ranking category-selective regions by their verticality bias (mean *y*-coordinate of pRF centers) reveals an additional categorical split, regions cluster together with the largest preference for the lower visual field in limb-selective regions, strongest in the OTS-limb region, followed by a moderate lower bias in face-selective regions, and lastly CoS-place with the least vertical bias (Fig. 5*f*).

### A single eccentricity gradient is not sufficient to explain category-selective pRF properties

If previous observations relating object-selective regions to retinal eccentricity biases consistently demonstrated that lateral VTC has a bias for sampling central visual space (Levy et al., 2001; Hasson et al., 2002; Malach et al., 2002; Kay et al., 2015; Gomez et al., 2019a; Poltoratski et al., 2021), then why does the limb-selective region located in lateral VTC show pRFs that are peripherally biased compared with face-selective cortex? While the limb-selective pRFs are consistent with the way limbs are visually experienced, how can we make sense of a peripherally biased category-selective region in a cortical location theorized to have a central bias? Fortunately, the same data offer a parsimonious solution for extending the original idea that category selectivity and retinal eccentricity input should be related while updating our characterization of functional organization of high-level vision. From the perspective of the lateral fusiform gyrus, receptive fields increase in size and eccentricity when one travels not only medially along the cortical surface, but also laterally. Does this suggest that the medial-lateral eccentricity gradient in VTC shows a reversal in lateral VTC, with two eccentricity gradients emanating from the lateral fusiform gyrus (FG)—one medially and one laterally?

**Figure 6.**
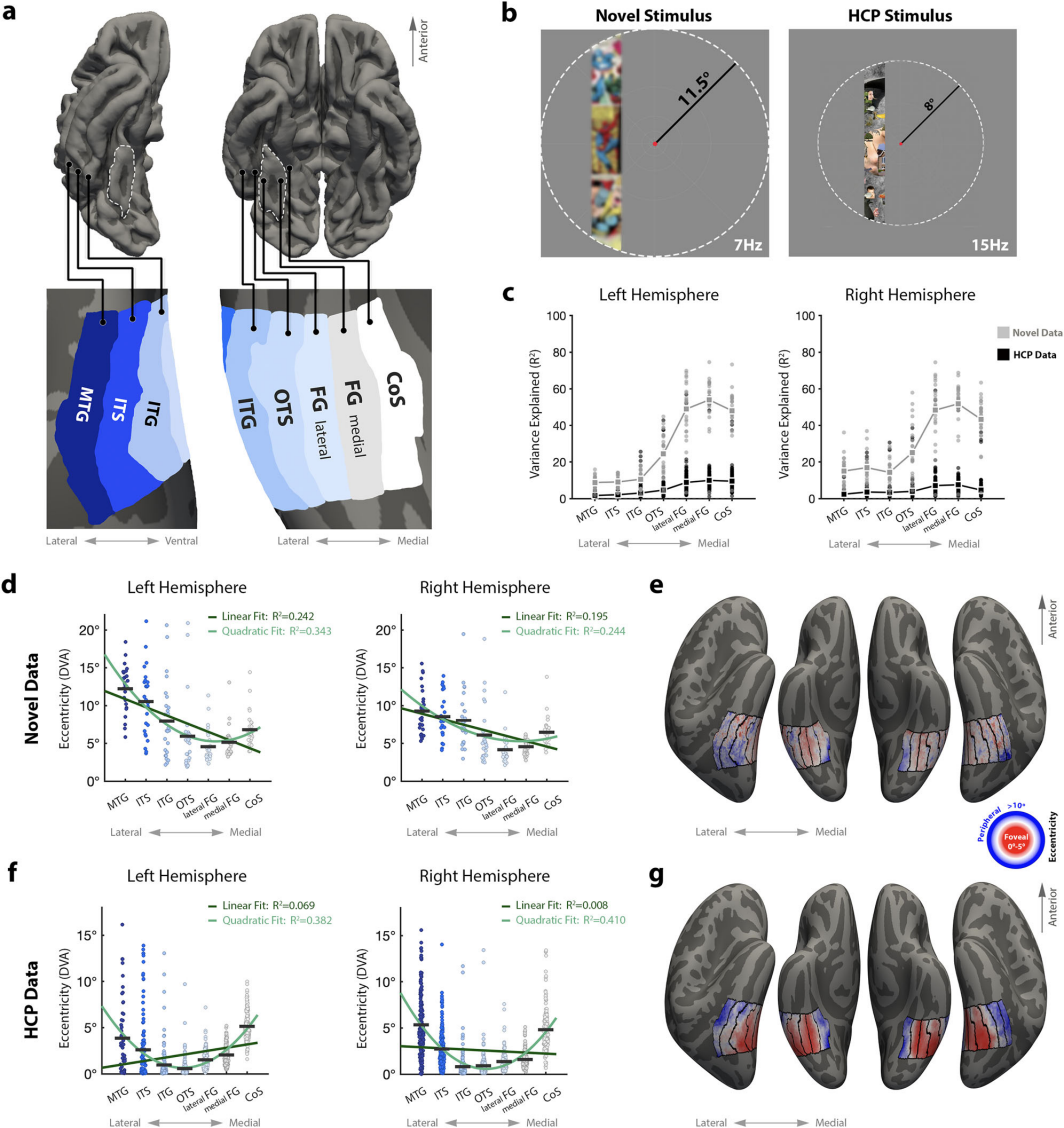
An eccentricity gradient reversal across ventral and lateral high-level visual cortex. ***a***, Labels in fsaverage space separate the HLVC into ROIs based on anatomical landmarks along the medial-lateral axis. Top row shows pial surface view, bottom row zooms into labeled regions on an inflated surface for clarity. Fusiform gyrus marked in dashed white line on pial surface for reference. ***b***, Stimuli for the novel pRF mapping experiment (left) and the HCP experiment (right). Note differences in stimulus content, radius (11.5 vs 8° of visual angle) and refresh rate (7 vs 15 Hz). Fixation point enlarged for visibility. Image within bar blurred in this figure to adhere to copyright policy. ***c****,* Variance explained in each anatomical ROI. Dots represent data from a single participant in the novel experiment (gray) and HCP experiment (black). Squares mark group means, connected by lines. ***d****,* Swarm plots depicting eccentricity values (in degrees of visual angle; DVA) across the medial-lateral axis from 27 participants. Dots represent average eccentricity from a single participant in a single ROI. Black horizontal lines represent mean eccentricity per ROI across participants. Dark green line shows fit of linear model, light green line shows fit of quadratic model. ***e***, Average eccentricity maps (*n* = 27 participants) in the ROIs defined in ***a***, displayed on an inflated cortical surface of the FreeSurfer average brain in an inferior view. Each vertex is colored according to the average pRF model-fit eccentricity value. Warmer colors indicate lower eccentricity (more foveal) while cooler colors represent preference for more eccentric (peripheral) stimuli. Note the warmer colors around the fusiform gyrus, and cooler colors in the CoS and MTG. ***f***, Swarm plots depicting the same analysis performed in ***d*** on the HCP 7T retinotopy dataset (*n* = 181). ***g***, Average eccentricity maps as described in ***e*** from the HCP 7T retinotopy dataset (*n* = 181). Labels, from medial to lateral: CoS, collateral sulcus; FG medial, medial fusiform gyrus; FG lateral, lateral fusiform gyrus; OTS, occipital-temporal sulcus; ITG, inferior temporal gyrus; ITS, inferior temporal sulcus; MTG, middle temporal gyrus.

To test this hypothesis in an observer-independent fashion, we parcellated visual cortex into seven anatomically defined ROIs according to cortical folds, with the medial-most ROI defined by the collateral sulcus and the lateral-most ROI defined by the middle temporal gyrus (Fig. 6*a*). Anatomical ROIs were drawn in a common cortical space (i.e., fsaverage), and all participants’ eccentricity maps were transformed into this space using cortex-based alignment. We thresholded voxels (*R*^2^ > 0.1) and calculated the mean eccentricity of pRFs within each anatomical ROI, for each participant. When the mean eccentricity values of each ROI are plotted in order along the lateral to medial axis, a clear U-shaped gradient emerges with pRFs in the most lateral and medial ROIs demonstrating higher eccentricity values than those located near the middle, around the fusiform gyrus (Fig. 6*d*). A repeated-measures (within-subject) ANOVA indicated that eccentricity values differ significantly across anatomical ROIs, in both hemispheres (right: *F*_(6)_ = 18.3, *p* < 0.001; left: *F*_(6)_ = 26.46, *p* < 0.001). To better understand the pattern of change in eccentricity values along the medial-lateral axis, we performed a model comparison analysis, fitting the data with both a linear model and a quadratic model and comparing the goodness of fit between those models. We found that the fit of the quadratic model was better than that of the linear model with higher variance explained and lower root mean square error in both hemispheres (right-linear: $R^{2}=0.19,\;\;\mathrm{RMSE}=2.96$, right-quadratic: $R^{2}=0.24,\;\mathrm{RMSE}=2.87$; left-linear: $R^{2}=0.24$, RMSE=3.85, left-quadratic: $R^{2}=0.34,\;\;\mathrm{RMSE}=3.6$). These data provide quantitative support for a U-shaped gradient which reverses sign in lateral ventral temporal cortex.

To test the robustness of this finding, we used data from the HCP 7T Retinotopy Dataset (N. C. Benson et al., 2018) which employed different stimulus properties, mapping techniques, and a higher field-strength fMRI. A comparison of stimulus properties and variance explained between the datasets is shown in Figure 6*b*,*c*. We repeated our analysis to test if our results would replicate in this larger (*n* = 181), independent sample. With both datasets in fsaverage space, we could use the same anatomical labels to repeat our previous analysis on this independent data. We find strikingly similar and significant results: the eccentricity values are lowest (most foveal) in lateral ventral temporal cortex and become more eccentric with increasing distance from the FG either medially or laterally (Fig. 6*f*,*g*). We ran a within-subject, repeated-measures ANOVA and found that in both hemispheres, eccentricity values differ significantly across anatomical ROIs (right: *F*_(6)_ = 107.8, *p* < 0.001; left: *F*_(6)_ = 21.96, *p* < 0.001). In the model comparison analysis, we found that the goodness of fit of the quadratic model was much higher than that of the linear model in both hemispheres (right-linear: $R^{2}=0.008,\;\mathrm{RMSE}=2.63$, right-quadratic: $R^{2}=0.41$, RMSE=2.03; left-linear: $R^{2}=0.07,\;\mathrm{RMSE}=2.4$, left-quadratic: $R^{2}=0.382,\;\mathrm{RMSE}=1.96$). Comparing the spatial distribution of variance explained between the datasets, we found especially low values of variance explained in the lateral regions in the HCP data, which could cause preferential exclusion of peripheral pRF fits (Fig. 6*c*). We therefore avoid drawing fine-grained conclusions about differences in the location of the trough between datasets and focus on the general gradient reversal pattern across the medial-lateral axis shared by both datasets. Taken together, these findings offer evidence for a functional topography in which the representation of retinal eccentricity reverses, suggesting that it is a general, replicable organizational principle across ventral and lateral visual streams.

## Discussion

Through a combination of naturalistic eye-tracking and population receptive field mapping, we find that limb and body stimuli are sampled with relatively peripheral and lower portions of the retina compared with faces, mirroring the peripheral receptive field coverage of limb-selective regions. By mapping receptive fields across ventral and lateral temporal cortex, we reveal a U-shaped retinal eccentricity gradient which reverses in sign near the lateral fusiform gyrus, with eccentricity biases becoming increasingly peripheral as one travels either medially or further laterally from the lateral fusiform. This updated model of functional topography in high-level visual cortex offers evidence for a parsimonious, larger-scale organizational pattern of visual cortex.

### An eccentricity gradient reversal explains the peripherally biased pRFs of the ventral limb-selective region

The first evidence for a cortical region that responds selectively to hands was observed in single cell recordings in macaque IT cortex (Gross et al., 1969, 1972). This groundbreaking discovery of selectivity to such high-level categories was not immediately accepted, with the first replication published a full decade later (Richmond et al., 1983). In the early 2000s, fMRI studies of the responses to body stimuli lead to the novel definition of the extrastriate body area (EBA), a large region in the lateral occipitotemporal cortex (LOTC), located near the motion-sensitive MT cortex (Downing et al., 2001). Follow-up fMRI studies have revealed additional regions identified near the mid-fusiform gyrus (mFG; Peelen and Downing, 2005; Schwarzlose et al., 2005), as well as in the posterior superior temporal sulcus (pSTS; Pinsk et al., 2009), later being parcellated more finely according to their anatomical locations (Weiner et al., 2010; Weiner and Grill-Spector, 2011). While the current work uses hand stimuli to define limb-selective regions, previous work has demonstrated that different limb (hands vs feet) or body stimuli (whole body vs limb) elicit overlapping but spatially distinct representations (Bracci et al., 2010; Bracci and Peelen, 2013). Future work can specify whether hands and feet, which may have different viewing patterns, map onto even finer-scale eccentricity bias differences within the OTS-limb region. Despite sharing the same consistent relationship to cortical folding as face- and place-selective regions, limb-selective regions were never formally included in models describing this consistent relationship between category selectively and cortical folding.

This relationship between category selectivity and the topography of retinal eccentricity is thought to scaffold the consistent organization of high-level visual cortex across individuals, whereby each region’s location is anchored to a particular cortical fold (Weiner et al., 2014; Grill Spector et al., 2017). It is posited that objects viewed with central vision lead to specialized clusters in the cortex with foveal retinal input, and stimuli viewed with peripheral vision in the cortex with more connectivity to the retinal periphery (Levy et al., 2001; Hasson et al., 2002; Gomez et al., 2019a). The initial focus on face- and place-selective cortex led to the idea of a medial-lateral eccentricity gradient in ventral temporal cortex, with medial VTC showing more input from the visual periphery and lateral VTC showing a bias for central visual space. If this gradient continues uninterrupted into the lateral temporal surface, this would predict that the limb-selective region in the OTS, abutting face-selective cortex laterally, should also show a bias for central visual space. However, our observation that limb-selective pRFs in VTC are relatively peripheral compared with face-selective cortex suggests a single medial-lateral eccentricity gradient is not an optimal model of VTC functional organization. Instead, we find evidence for a reversal of the medial-lateral eccentricity gradient near the lateral fusiform gyrus, with the cortex that is increasingly lateral to VTC showing increasing bias for sampling peripheral visual space. This reversal can be seen whether measured through functional (Fig. 4*f*) or broader anatomical ROIs (Fig. 6*d*). This new model can explain both the peripheral pRFs of the OTS-limb region as well as the general peripheral bias observed in the lateral visual stream. This primary description of mapped receptive fields supports a novel organization principle of high-level visual cortex in which the ventral and lateral streams of visual processing may not be as separate as once described, but instead linked by a shared and diverging gradient of receptive field properties, similar to tuning for higher-level features such as real-world object size (Konkle and Caramazza, 2013).

Despite being one of the last category-selective regions to have its RF properties described, the limb-selective region of the OTS is influential given that its peripherally biased pRFs necessitated a rethinking of high-level visual cortex topography. The current theory describing the category-selective regions of the ventral temporal lobe was rooted in the notion that axonal connectivity from early retinotopic maps creates an input bias, with the lateral regions receiving foveal input and medial ones, peripheral input. By extending recent observations that a face-selective region on the lateral temporal surface shows strong peripheral connectivity (Pitcher et al., 2020; Finzi et al., 2021), we propose an extension of the current characterization of eccentricity bias in VTC. Cortical regions beyond the lateral fusiform show a reversal for preferred retinotopic input and sample from increasingly peripheral locations of the retinal image, ultimately forming a diverging gradient between the collateral sulcus of the medial ventral temporal lobe and the middle temporal gyrus of the lateral temporal lobe. Note that this gradient is a broad pattern of functional topography analyzed at the scale of large anatomical regions (Fig. 6); future work should characterize the finer-scale variability within a given anatomical region.

The idea that there are two increasing eccentricity gradients sharing a common foveal representation is consistent with previous observations that visual field maps share a border at a reversal of polar angle gradients (Sereno et al., 1995; Wandell et al., 2007). The existence of multiple, discrete foveal representations shared by clusters of visual field maps across visual cortex (Wandell et al., 2007) necessarily implies that eccentricity representations should also show such reversals. Indeed, studies of ventral visual cortex have identified an eccentricity reversal on the border separating the human V4 (hV4) and “ventral occipital” 1 (VO-1) field maps, perpendicular to the direction of the polar angle gradient (Brewer et al., 2005; Winawer and Witthoft, 2015). Another such reversal was reported separating these VO field maps from more anterior maps in parahippocampal cortex (Arcaro et al., 2009). The eccentricity gradient reversal we describe shares the foveal representations of the VO1-2 and PHC1-2 field map clusters (Brewer et al., 2005; Arcaro et al., 2009; Winawer and Witthoft, 2015) and is thus oriented medial-lateral, perpendicular to the direction of phase-map reversals of the ventral visual stream field maps (Wang et al., 2015). In this case, the reversal of an eccentricity gradient, which seems to happen over larger swaths of cortex, might represent broader-scale functional transitions, such as separation points between the ventral, lateral, and dorsal streams of visual processing (Goodale and Milner, 1992; Ungerleider and Haxby, 1994; Pitcher and Ungerleider, 2021; Weiner and Gomez, 2021).

### Peripherally biased receptive fields in limb-selective regions reflect the viewing statistics of body stimuli

Using a receptive field mapping technique designed for driving reliable responses in HLVC, we demonstrate that pRFs in limb-selective regions are biased toward the lower periphery of the visual field. With naturalistic eye-tracking, we find that humans fixate on faces regardless of their size or position, statistically placing bodies and limbs in the periphery of the lower visual field. In line with previous studies (Kay et al., 2015; Gomez et al., 2018; Finzi et al., 2021; Poltoratski et al., 2021), we find that pRFs in face-selective cortex are biased toward the horizontal meridian, consistent with previous reports suggesting that the tendency to fixate on and between the eyes compared with the mouth leads to this horizontal sampling bias (Fedor et al., 2018; Gomez et al., 2018; Masulli et al., 2022). Although previous work has demonstrated that laboratory-based eye-tracking metrics, especially on faces, generalize to real-world contexts (Peterson et al., 2016), this could be further tested using glass-type eye-tracking devices and a camera to test whether this naturalistic behavior replicates in real-life environments.

Our findings suggest that biases in spatial computations of high-level vision mirror our visual experience. This is largely consistent with previous work showing that limb-selective cortex in VTC is initially large in surface area in younger children and is recycled with development, overtaken by the growing representations for faces and words (Nordt et al., 2021). This cortical recycling is thought to occur as a result of a transition from a bias in toddlers toward looking at hands (Frank et al., 2012; Fausey et al., 2016), to heavier visual sampling of faces and words in school-age children (Dehaene-Lambertz et al., 2018; Nordt et al., 2021). This might suggest that in toddlers who have not yet learned to read, limb-selective cortex may show a larger bias for sampling central visual space.

Previous work measuring neural responses demonstrated that social stimuli including faces and bodies elicit the largest responses when presented in their typically experienced orientations (Chan et al., 2010). Coupled with observations showing that faces are powerful attractors of attention and eye gaze (Cerf et al., 2009; Yarbus, 2013) and make up a dense proportion of central visual input within the first few months of life (Jayaraman et al., 2017), all suggest that bodies and limbs, as visual stimuli, largely enter the visual system through the periphery of the retinal image. This matches well our empirical observation that adults place body and limb stimuli in peripheral locations of the retina relative to face stimuli. This peripheral retinal sampling also mirrors data from our pRF model fits, where we find that limb-selective pRFs were significantly more eccentric than the centrally biased fits of face-selective cortex (Figs. 4, 5). While pRFs of the OTS-limb region do not extend as peripherally as those of the CoS-place region (Fig. 4), this could be a result of scene and place stimuli being larger in real-world and retinal size than limbs. It should also be noted that the limb-selective regions on the lateral occipitotemporal surface, compared with the ventral region, show larger and more peripheral receptive fields; an observation for which the current behavioral and neural measures cannot account. Limb-selective regions on the lateral surface abut and overlap motion-selective cortex (Weiner and Grill-Spector, 2011), consistent with the higher preference for dynamic stimuli in lateral category-selective regions (Pitcher et al., 2011). It is this preference for moving visual stimuli that may necessitate larger receptive fields to pool information over a larger expanse of space to compute an object’s trajectory, which would be consistent with developmental work showing that pRFs in motion-selective cortex become larger and sample more of peripheral visual space into adulthood (Gomez et al., 2019b).

### Nonsensory connectivity likely plays a role in determining the cortical location of the limb-selective region

While this expanded functional organization is likely inherited from axonal wiring established during gestation (Kamps et al., 2020), it does not have to be the only feature that determines the cortical location and properties of a category-selective region, given that these category regions receive not only axonal input from earlier visual cortex (e.g., via inferior longitudinal pathways; Tusa and Ungerleider, 1985) but receive and contribute axons to the vertical occipital fasciculi (VOF; Yeatman et al., 2014; Takemura et al., 2016) and the arcuate fasciculi (AF; Catani and Thiebaut de Schotten, 2008). Indeed, the area around the OTS appears to be a transition zone: despite limb-selective cortex, word-selective cortex, and potentially other learned categories like Pokémon all cohabitating this cortical fold, words (Le et al., 2017) and Pokémon (Gomez et al., 2019a) show foveally biased pRF sampling. For example, word-selective cortex and thus its receptive fields likely depend on language lateralization (Cai et al., 2010; Dundas et al., 2013) rather than purely visual processes alone. Thus, while the eccentricity gradient reversal seems to be a broad cortical feature that plays a role in the gross functional organization in high-level vision, it is likely not the only feature contributing to pRF properties. When one considers that the eccentricity gradient we observe shows a reversal at the lateral fusiform gyrus, this necessarily means that there are relatively peripheral representations both lateral and medial to this transition point. The fact that the ventral limb-selective region emerges laterally to face-selective cortex and not medially suggests the existence of other factors underlying the topographic organization of high-level vision. Attention, for example, is another top-down factor that plays a role in scaling visual responses in ventral visual cortex (Bugatus et al., 2015) and can shift the spatial properties of receptive fields (Sprague and Serences, 2013; Kay et al., 2015). In the current dataset, participants were instructed to fixate on the center but attend to the sweeping bar, whereas in the HCP dataset (N. C. Benson and Winawer, 2018), they were attending centrally. Attention-related information is communicated in part from dorsal parietal cortex to ventral temporal cortex via the VOF, which shows a connectivity bias toward lateral VTC (Takemura et al., 2016). This attentional difference may partially explain some differences between the datasets but nonetheless demonstrates that the eccentricity gradient we present here is robust to changes in attention.
